# Preoperative assessment and optimisation prior to planned aortic aneurysm repair: a UK survey examining current practice and attitudes of vascular surgeons and vascular anaesthetists

**DOI:** 10.1186/s13741-023-00304-4

**Published:** 2023-06-13

**Authors:** Phoebe Scarfield, Jack Ryan, Morad Sallam, Athanasios Saratzis, Adam C. Pichel, Jugdeep K. Dhesi, Judith S. L. Partridge

**Affiliations:** 1 Guy’s and St. Thomas’ NHS Foundation Trust, London, SE1 4YB UK; 2 NIHR Leicester Biomedical Research Centre GB, Leicester, LE5 4PW UK; 3 Manchester Royal Infirmary, Manchester, M13 9WL UK

**Keywords:** Multidisciplinary team, Preoperative assessment, Perioperative medicine, Aortic aneurysm repair, Frailty, Shared decision-making, POPS, Older people, Surgery, Prehabilitation

## Abstract

**Background:**

The majority of those diagnosed with aortic aneurysm in the UK are older, multi-morbid patients. Decision-making as to who may benefit from intervention (open or endovascular aneurysm repair) is highly variable across the NHS (as is the mode of intervention), in part because there are no detailed guidelines or consensus on preoperative assessment. Thus, there is likely to be significant variation in the pre-operative assessment and optimisation of these patients.

**Methods:**

A survey was designed to understand current practice and attitudes of vascular surgeons and vascular anaesthetists in the UK regarding preoperative assessment and optimisation of patients undergoing elective aortic aneurysm repair. The survey was reviewed and validated by an expert panel, then distributed electronically to all vascular surgical and vascular anaesthetic leads in the UK.

**Results:**

Overall, the response rate was 68%. The responses were varied between surgeons and anaesthetists, with differences reported in the preoperative assessment and optimisation of patients, the approach to shared decision-making, and the perioperative pathway.

**Conclusions:**

Despite initiatives such as Getting It Right First Time (GIRFT) and National Institute for Health and Care Excellence (NICE) guidelines, variation still exists between centres with some differences in opinion observed between surgeons and anaesthetists. These differences may be leading to duplication of work in the perioperative pathway, inconsistencies in how risk is assessed and communicated with consequent variation in patient care. Addressing these issues requires awareness and implementation of existing guidelines, transdisciplinary working, efficient data-driven pathways, and structured aortic aneurysm multi-disciplinary team to promote meaningful shared decision-making.

## Background

In 2020, nearly 3000 patients underwent elective abdominal aortic aneurysm (AAA) repair in the United Kingdom. Of these patients, 94% with infra-renal aneurysms and 87% with complex aneurysms were aged over 65 years (Vascular Services Quality Improvement Programme (VSQIP) 2021). Many older patients may previously have been deemed too high risk for open repair, but now undergo endovascular surgery (Mastracci et al. 2015). Whilst this increased access to aneurysm surgery for older patients may be appropriate, the coexistence of multimorbidity and frailty in older patients presents specific anaesthetic and medical challenges, and necessitates adaptation of perioperative pathways to meet these specific clinical needs (Vascular Services Quality Improvement Programme (VSQIP) 2021; Pearse et al. 2006). Patient-centred pathways have evolved, but inconsistently, with resultant variation in perioperative management of patients with aortic aneurysm, both within and between centres (Partridge et al. 2015). Such variation is particularly observed in preoperative assessment, investigation and optimisation across different domains, including cardiac status, respiratory fitness, frailty and anaemia. Furthermore, whilst patients with AAA frequently have undiagnosed cognitive impairment and frailty, preoperative assessment and optimisation of these issues is rarely routine, with potential impact on length of hospital stay (LOS) (Partridge et al. 2014; Partridge et al. 2015). Such variation in the perioperative care of patients with aortic aneurysms may exist due to a paucity of clinically relevant evidence and/or inconsistent implementation of perioperative guidelines (GIRFT 2022). To address these issues, the Abdominal Aortic Aneurysm Quality Improvement Programme (AAA QIP) was established in the United Kingdom with the following aims: to identify high-risk individuals, provide a pathway for those not proceeding with aneurysm surgery, involve vascular anaesthetists in decision to treat, and inform patient choice (Abdominal Aortic Aneurysm Quality Improvement Programme (2012). However, this quality improvement programme did not offer detailed recommendations for preoperative assessment processes or advise specific preoperative investigations. With the centralisation of vascular services there is a need to standardise preoperative assessment and optimisation processes thereby reducing variation in practice with potential benefits for patients and clinical services through improved efficiency and reduced cost. This survey aims to understand current practice and attitudes of vascular surgical and anaesthetic leads in the UK.

## Methods

A survey was designed to explore the current practices and attitudes of surgeons and anaesthetists regarding perioperative pathways of care for patients undergoing elective aortic aneurysm repair. The survey content was informed by themes from the AAA QIP and Royal College of Anaesthetists perioperative programme. Survey design involved a combination of multiple choice, ranking and Likert formats. An expert panel reviewed the survey to ensure readability, non-ambiguity and content validity with a validation score of + 0.56 (Lawshe 1975). The survey was distributed electronically using web-based Survey Monkey software. To maximise response rate, the survey was endorsed and jointly conducted by the Vascular Society of Great Britain and Ireland (VS) and the Vascular Anaesthesia Society of Great Britain and Ireland (VASGBI) (The Vascular Society of Great Britain Ireland 2022; VASGBI 2022). The survey was distributed to leads in vascular surgery and anaesthesia at all UK trusts via email with reminders sent over an 8-week period (2, 4, and 6 weeks). Responses were analysed using Microsoft Excel and free-text answers were grouped according to themes and reported descriptively. The Health Research Authority (HRA) ethics tool (NHS Health Research Authority, Medical Research Council. Do I Need NHS Research Ethics Committee Approval Decision Tool. Available from: hra-decisiontools.org.uk/ethics/ 2022) was used which advised that no research ethics approval was necessary. Participation was voluntary and patients were not involved. All data was anonymously collected and stored securely.

## Results

The survey was sent to 75 vascular surgeons and 73 vascular anaesthetists. Responses were received from 45 vascular surgeons and 48 vascular anaesthetists giving a response rate of 64% across both groups. These were received from 17/18 regions of the UK. Surgeons responding to the survey reported an average of seven vascular consultants performing elective surgical lists at their centre (range 4–14). Anaesthetists responding reported an average of eight vascular anaesthetists regularly working on elective lists (range 0–18) with 39 (81%) anaesthetists stating that they have a named clinical lead for vascular anaesthesia at their centre. Anaesthetists reported working across a range of surgical specialties with no sole vascular anaesthesia appointments. The annual number of elective aneurysm repairs varied between centres with higher volumes of standard EVAR compared to FEVAR and open aneurysm repairs.

### Preoperative assessment

Sixty-eight (73%) of respondents reported that preoperative assessment occurs at the hub site with twenty (22%) percent reporting preoperative assessment at both the hub and spoke sites. Six (6%) respondents reported preoperative assessment occurred solely at the spoke site.

Averaged across all surgery types, 75% (*n* = 30) of vascular surgeons reported patients were routinely preoperatively assessed by vascular surgeons. In addition, surgeons reported that patients were preoperatively assessed in either general (55%, *n* = 22) or vascular specific (41%, *n* = 17) nurse led preoperative assessment clinics. Surgeons reported that anaesthetists preoperatively assessed a greater number of patients undergoing FEVAR (94%, *n* = 31) or open repair (95%, *n* = 39) compared to standard EVAR surgery (80%, *n* = 35).

**Fig. 1:**
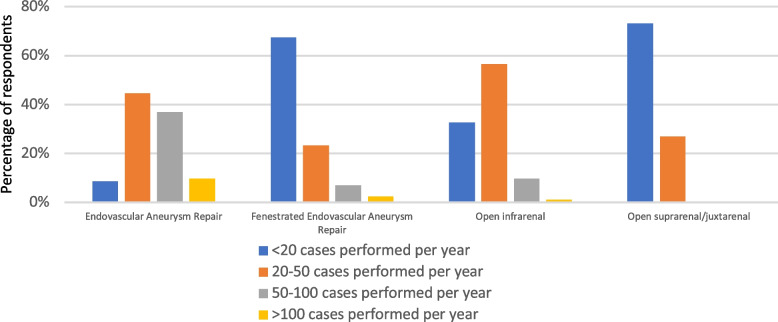
Annual volume of cases according to procedure type

**Fig. 2:**
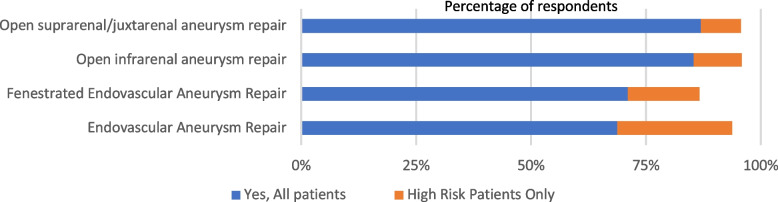
Percentage of vascular anaesthetists who report preoperative assessment of all patients vs high risk patients only, according to surgical type

Vascular anaesthetists reported routinely preoperatively assessing patients undergoing FEVAR (71%, *n* = 32), open suprarenal/juxtarenal repair (87%, *n* = 40), open infrarenal (85%, *n* = 41) and standard EVAR (69%, *n* = 33). Twelve (25%) anaesthetists reported preoperatively assessing patients undergoing EVAR only if they were deemed high risk (Figs. 1 and 2). Thirty (63%) anaesthetists reported patients were identified as high-risk at vascular MDT, 28 (58%) at surgical consultation, 22 (45%) at nurse led preoperative assessment, 21 (44%) at anaesthetic review, or 5 (10%) on referral documentation from hub site. One free-text response stated that patients ‘undergo Cardio-Pulmonary Exercise Testing (CPET) and are then identified as high risk.’

Twenty-nine percent (*n* = 13) of surgeons reported using a perioperative risk score compared to 63% (*n* = 30) of anaesthetists. Scores included Carlisle risk calculator, V-POSSUM, SORT, and AS-NSQIP (Carlisle et al. 2015; Risk Prediction in Surgery 2022; Protopapa et al. 2014; College and of Surgeons. ACS NSQIP surgical risk calculator. 2022). Free-text responses highlighted that often multiple scores are used for different patient cohorts depending on perceived risk. Not all respondents outlined which score they use. Averaged across all surgery types, the Vascular Society Safe for Intervention Checklist was reported to be used routinely by 28% (*n* = 12) of surgeons and 20% (*n* = 9) of anaesthetists (College and of Surgeons. ACS NSQIP surgical risk calculator. 2022). Thirty-two percent (*n* = 13) of surgeons and 19% (*n* = 8) anaesthetists agree the checklist is a useful tool for the pre-operative work up of patients undergoing aneurysm repair. Notably, 62% (*n* = 27) of surgeons and 73% (*n* = 35) of anaesthetists neither agreed nor disagreed about the usefulness of the checklist. Both surgeons and anaesthetists reported that CPET was the most common preoperative investigation routinely requested for patients undergoing all types of aneurysm repair (Table 1). Averaged across all surgical types, a slightly higher proportion of surgeons (58%) reported that Transthoracic Echocardiogram (TTE) was routinely performed compared to anaesthetists (42%). No respondent reported undertaking Cardiac MRI in the routine preoperative assessment of patients undergoing aneurysm repair. Myocardial Perfusion Scan (MPS) and Stress Echocardiograms were rarely requested routinely. It was noted in free-text comments that practice changed during the COVID-19 pandemic due to restricted availability of certain services. For example, if CPET was not available, there was a greater reliance on pulmonary function tests and TTE. Free-text comments also stated that N-terminal pro Brain Natriuretic peptide (NT pro-BNP) is checked routinely, with further investigations requested only of levels are raised. Surgeons (52%, *n* = 22) and anaesthetists (56%, *n* = 27) agreed that national guidance on preoperative investigation is required. Surgeons reported a need for national guidance on preoperative investigation for open aneurysm repair (57.6%, n = 25), for EVAR (44.2%, *n* = 19) and FEVAR (48.8%, *n* = 20). The majority of anaesthetists agree that national guidance would be helpful across all surgical procedures (56%, *n* = 27).

**Table 1:** Routine preoperative investigations according to surgical procedure

	Endovascular aneurysm repair		Fenestrated endovascular aneurysm repair		Open infrarenal		Open suprarenal	
	Anaesthetics	Surgeons	Anaesthetics	Surgeons	Anaesthetics	Surgeons	Anaesthetics	Surgeons
Transthoracic echocardiogram	42.11%	58.54%	42.11%	58.82%	42.22%	59.52%	41.86%	57.89%
Myocardial perfusion scan	7.89%	4.88%	7.89%	5.88%	11.11%	4.76%	13.95%	10.53%
Stress Echocardiogram	2.63%	2.44%	7.89%	5.88%	11.11%	11.90%	18.60%	13.16%
Pulmonary function tests	47.37%	56.10%	39.47%	55.88%	44.44%	54.76%	41.86%	50.00%
Cardio-pulmonary exercise testing	65.79%	65.85%	65.79%	76.47%	68.89%	78.57%	72.09%	78.95%

### Comorbidity and optimisation

**Fig. 3:**
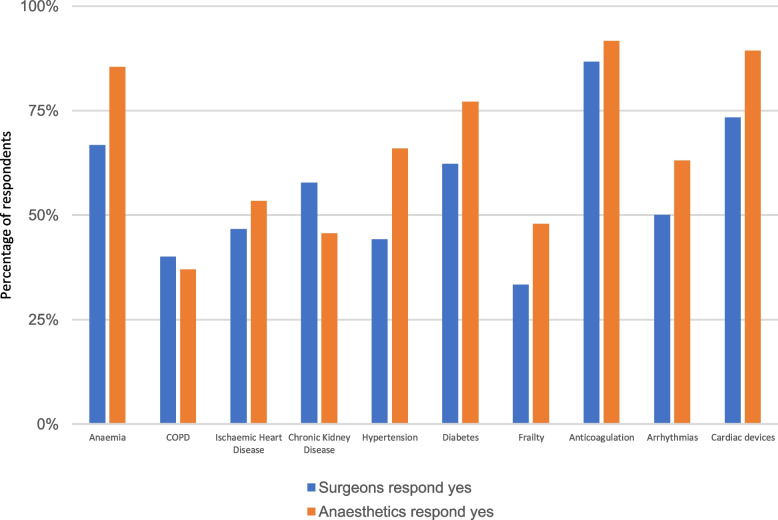
Proportion of respondents who report an existing pre-operative pathway or guideline for the assessment and optimisation of the following conditions

Figure 3 reports surgeon and anaesthetist awareness of condition specific perioperative guidelines in existence at the trust in which they work. The medical issues reported as having the fewest hospital guidelines and pathways were frailty (39%, *n* = 36) and COPD (38%, *n* = 35).

There were differences between surgeons and anaesthetists when asked who they referred to for the optimisation of co-morbidities. Vascular surgeons reported referral to organ-specific physicians except for frailty where 33% (*n* = 15) reported referring to geriatricians with an interest in perioperative medicine. Anaesthetists reported referring to organ specific physicians for most conditions except anaemia where 48% (*n* = 23) referred for further anaesthetics-led anaemia management, and frailty where 33% (*n* = 16) reported referring to geriatricians with an interest in perioperative medicine.

**Fig. 4:**
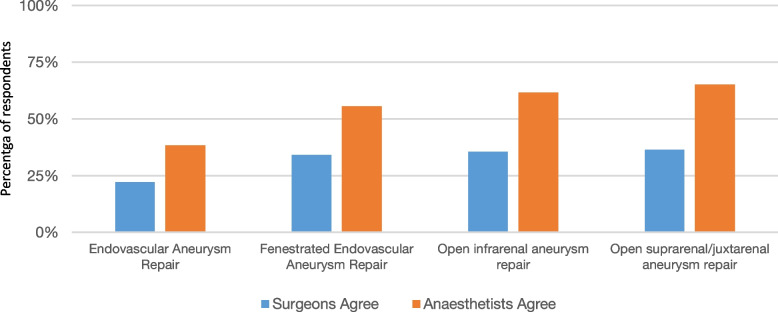
Percentage of respondents who agree with the statement ‘all patients should be seen by a peri-operative physician before surgery’

Averaged across all surgery types, 18% (*n* = 8) of vascular surgeons agreed with the statement ‘vascular anaesthetists are not trained to optimise patients prior to planned surgery.’ In comparison, 30% (*n* = 14) of vascular anaesthetists agreed with this statement. Averaged across all surgery types, both surgeons (66%, *n* = 29) and anaesthetists (78%, *n* = 37) disagreed or strongly disagreed that ‘there is no role for geriatricians with an interest in perioperative medicine’. A greater proportion of anaesthetists agreed that all patients should be seen by a perioperative physician, especially for open aneurysm repair (Fig. 4).

### Shared decision-making

The responses to the question, ‘please rate how important you feel the following issues are when communicating with patients as part of shared decision-making’, differed between surgeons and anaesthetists (Table 2). Both surgeons (80%, *n* = 36) and anaesthetists (85%, *n* = 41) think it is important to discuss 12-month mortality following surgery, yet 49% (*n* = 22) of surgeons and 42% (*n* = 20) of anaesthetists reported routinely discussing 12-month mortality. Similarly, 58% (*n* = 26) of surgeons and 77% (*n* = 37) of anaesthetists reported the importance of discussing risk of postoperative delirium, yet 27% (*n* = 12) of surgeons and 56% (*n* = 27) of anaesthetists reported discussing it routinely. If there were areas that respondents wanted to discuss as part of shared decision-making but felt unable, they were asked to identify barriers to discussion. Fifty-eight percent (*n* = 18) of surgeons who responded reported that the lack of geriatricians with perioperative interest was a barrier to shared decision-making. Additional barriers to shared decision-making noted in the free-text comments by anaesthetists included 'no job planned anaesthetics attendance at MDT’, ‘lack of accurate/reliable information’, 'NICE guidelines at odds with current practice, but no direction on how to assess patients.’ One surgical free-text response commented ‘lack of robust tools to predict certain outcomes.’

**Table 2:** Percentage of respondents; ‘Please rate how important you feel the following issues are when communicating with patients as part of shared decision-making.’

	Anaesthetics		Surgeons	
	Think important	Routinely discuss	Think important	Routinely discuss
Estimated life expectancy with surgery	93.75%	77.08%	100.00%	82.22%
Estimated life expectancy without surgery	93.75%	80.85%	100.00%	83.72%
30-day mortality following surgery	87.50%	87.23%	100.00%	97.78%
12-month mortality following surgery	87.24%	42.55%	81.82%	48.89%
Risk of re-intervention	87.24%	66.67%	97.78%	100.00%
Risk from general anaesthetic	79.17%	83.33%	84.44%	75.00%
Risk from neuroaxial block	74.47%	75.00%	66.67%	37.78%
Length of stay in hospital	81.25%	83.33%	80.00%	93.33%
Perioperative cardiac risk	89.58%	91.67%	95.55%	95.56%
Risk of acute kidney injury	89.58%	81.25%	93.33%	93.33%
Risk of dialysis	91.67%	66.67%	93.34%	77.78%
Risk of venous thromboembolism	65.96%	48.94%	82.22%	73.33%
Risk of delirium	78.72%	56.25%	57.77%	27.27%
Risk of functional deterioration postoperatively	97.92%	89.58%	95.56%	95.56%

### Supporting pathways and data management

When asked if there was an enhanced recovery pathway in their unit, there was a difference in responses from surgeons and anaesthetists. Fourteen (29%) anaesthetists answered yes for EVAR, 5 (10%) for FEVAR, 10 (21%) for open infra-renal repair and 7 (15%) for open supra/juxta-renal repair. 26 (58%) surgeons answered yes for EVAR, 12 (27%) for FEVAR, 20 (44%) for open infra-renal repair and 14 (31%) for open supra/juxta-renal repair.

Regarding discharge planning, 14 (31%) surgeons responded affirmatively to the question, ‘is there an agreed protocol for the repatriation of patients to spoke site after undergoing planned aortic aneurysm repair at the hub site’ and 27 (60%) responded affirmatively to the question 'does the routine process for preoperatively assessing elective patients consider potential issues with hospital discharge?’.

Twenty-two (49%) surgeons report that data is kept on cases managed non-operatively. Free-text comments also state that ‘not specifically, but all cases are discussed at MDT so would be possible to find this data’ and one respondent who answered ‘no’ wrote 'but we should do’.

## Discussion

This is the first study to report attitudes and behaviours of UK vascular surgical and anaesthetic leads regarding pre-operative assessment of patients referred for aortic aneurysm repair. The responses to this survey highlight key themes that should be considered to improve the aortic aneurysm pathway of care. These include, but are not limited to, the variation and duplication of work in the peri-operative pathway, the inconsistencies in how risk is assessed and communicated, the reported lack of appropriate guidelines, job planning support for attendance at MDT, and the barriers to standardisation of clinical pathways.

### Pre-operative risk assessment

There was notable variation in pre-operative assessment of patients prior to aortic aneurysm repair. Approaches to pre-operative assessment varied according to surgery type and perceived level of perioperative risk. There was inconsistency in how and when patients were identified as high risk. In cases where risk stratification occurs late in the pathway, there is potential delay to investigation and optimisation. The NICE guidelines (NG156) on the diagnosis and management of abdominal aortic aneurysm published in 2020 (Guideline and (NG156). 2020) recommended that EVAR is indicated only for patients with hostile abdomens, medical comorbidities or anaesthetic risks that contraindicate open surgery. However, this guideline did not provide definitions for medical comorbidities or anaesthetic risk contraindicating open repair. This has potentially resulted in the continued variation observed in pre-operative assessment, optimisation and management of aortic aneurysm in this survey. As reported in this survey, the majority of pre-operative assessment is still undertaken in hub centres, and as such there is scope to implement standardised preoperative assessment and optimisation pathways across the UK vascular network.

### Optimisation

This survey also demonstrated variation in the availability and use of guidance and pathways for the perioperative management of medical conditions. In particular, the majority of responses indicated a lack of clear guidelines for the optimisation of frailty and COPD, two conditions known to affect a significant number of patients with aortic aneurysms (Partridge et al. 2017). Survey responses suggest differences between surgeons and anaesthetists in who they refer to for advice to optimise co-existent medical conditions. This variation may lead to unnecessary referrals to other specialties, with potential delay to surgery, inefficient use of resources and increased treatment burden for patients. For patients living with frailty, this may be addressed with implementation of recently published CPOC/BGS guidelines on perioperative management for patients living with frailty undergoing elective and emergency surgery (Centre for Perioperative Care. Guideline for Perioperative Care for People Living with Frailty Undergoing Elective and Emergency Surgery. 2021). Whilst specific perioperative guidelines may be required in certain conditions, in other situations application of disease specific guidelines (for example, NICE guideline COPD in over 16 s; diagnosis and management) should be employed by healthcare professionals involved in optimisation of patients for aneurysm repair (Institute and for Health and Care Excellence. NICE Guideline NG115. Chronic obstructive pulmonary disease in over 16s: diagnosis and management. 2018).

### Standardised approach to SDM

Respondents to this survey described difference in their attitudes and behaviours pertaining to shared decision-making. For example, the majority of respondents deemed it important to discuss delirium risk with patients, yet only a minority reported routinely discussing this. This may reflect a lack of confidence in identifying, modifying and discussing risk of postoperative delirium. This is in keeping with findings from a previous survey examining knowledge about delirium in surgical trainees (Shipway et al. 2015). The development of the new RCS core surgical training curriculum in the UK aims to address such gaps through inclusion of medical issues frequently encountered in the perioperative period including delirium (Programme et al. 2021). Further barriers to meaningful shared decision-making described in this survey included the ‘lack of robust tools to predict certain outcomes’, inadequate clinic time, or discussions occurring too late in the pathway. Different strategies to address this include; routine collection of objective measures of frailty and cognition (Partridge et al. 2014; Audit 2021); efficient outpatient services (College and of Physicians. Outpatients: the future – adding value through sustainability. 2018); early referral to perioperative medicine services for patients with sub-threshold or at-threshold aneurysms in order to facilitate timely preoperative assessment and optimisation (Partridge et al. 2017).

### Collaborative perioperative care

Interestingly, two thirds of vascular anaesthetists reported they were trained to optimise patients prior to surgery; however, most reported referring to other organ-specific specialties with the majority expressing a role for geriatricians with an interest in perioperative medicine. Transdisciplinary working aims to transcend traditional specialty boundaries and aim to share knowledge, skills and decision-making between specialties; a transdisciplinary team approach to aneurysm repair may address some of the issues highlighted by this survey through reducing barriers to shared decision-making, improving optimisation of multimorbidity, and streamlining use of hospital resources by avoiding unnecessary investigations or referrals. Such an approach requires multidomain assessment and optimisation using evidence-based guidelines and has been demonstrated to provide clinically and cost-effective perioperative care in elective vascular surgery (Partridge et al. 2017; Partridge et al. 2021).

### Limitations

The limitations of this survey are those inherent to all surveys. Questionnaire design and execution aimed to minimise the effects of response bias. As the survey was only distributed to vascular surgical and anaesthetic leads, these views may not be representative of all surgeons and anaesthetists working in each centre, especially relating to specific discussions about shared decision-making in the peri-operative period. These results will also have inherent reporting bias as we have asked peri-operative specialists about the importance of peri-operative pathways and care. However, responses were gained from 17 out of 18 regions across the UK suggesting a geographically representative sample. There is also the potential for ambiguity in responses from an anonymous survey as it is not possible to integrate responses from the same trust for the comparison of anaesthetist and surgeon opinion. However, the response rate for this survey was high for an online questionnaire (Colbert et al. 2013) and therefore the findings may provide a useful information with which to improve and standardise perioperative aneurysm management in the UK.

## Conclusion

This survey describes the attitudes and behaviours of vascular surgeons and anaesthetists regarding the preoperative assessment and optimisation of patients undergoing elective aortic aneurysm repair in the UK. Despite initiatives such as GIRFT and NICE guidelines, variation still exists between centres with some differences in opinion observed between anaesthetists and surgeons. Addressing these issues requires awareness and implementation of existing guidelines, transdisciplinary working between surgeons, anaesthetists, physicians and geriatricians with an interest in perioperative medicine, efficient data-driven pathways including timely referral for assessment and optimization, and structured aortic aneurysm MDT to promote meaningful shared decision-making. To successfully translate best practice into routine clinical care requires collaboration between specialist societies (for example the Vascular Society, Vascular Anaesthetists Society and the British Geriatrics Society in the United Kingdom) to produce a framework to support clinicians delivering care for patients with abdominal aortic aneurysms.
